# Dr. Daniel Maynard Burgess

**Published:** 1911-04

**Authors:** 


Dr. Daniel Maynard Burgess, for many years health inspector for the United States in Cuba and an authority on yellow fever, died at his home in New York, March 1, 1911, from pneumonia, aged 82 years.

